# Genome wide association and haplotype analyses for the crease depth trait in bread wheat (*Triticum aestivum* L.)

**DOI:** 10.3389/fpls.2023.1203253

**Published:** 2023-07-03

**Authors:** Chengxiang Song, Kaidi Xie, Xin Hu, Zhihua Zhou, Ankui Liu, Yuwei Zhang, Jiale Du, Jizeng Jia, Lifeng Gao, Hailiang Mao

**Affiliations:** ^1^ National Key Laboratory of Crop Genetic Improvement, Huazhong Agricultural University, Wuhan, China; ^2^ Institute of Crop Sciences, Chinese Academy of Agriculture Sciences, Beijing, China

**Keywords:** Cd, candidate genes, GWAS, selection sweep, wheat

## Abstract

Wheat grain has a complex structure that includes a crease on one side, and tissues within the crease region play an important role in nutrient transportation during wheat grain development. However, the genetic architecture of the crease region is still unclear. In this study, 413 global wheat accessions were resequenced and a method was developed for evaluating the phenotypic data of crease depth (CD). The CD values exhibited continuous and considerable large variation in the population, and the broad-sense heritability was 84.09%. CD was found to be positively correlated with grain-related traits and negatively with quality-related traits. Analysis of differentiation of traits between landraces and cultivars revealed that grain-related traits and CD were simultaneously improved during breeding improvement. Moreover, 2,150.8-Mb genetic segments were identified to fall within the selective sweeps between the landraces and cultivars; they contained some known functional genes for quality- and grain-related traits. Genome-wide association study (GWAS) was performed using around 10 million SNPs generated by genome resequencing and 551 significant SNPs and 18 QTLs were detected significantly associated with CD. Combined with cluster analysis of gene expression, haplotype analysis, and annotated information of candidate genes, two promising genes *TraesCS3D02G197700* and *TraesCS5A02G292900* were identified to potentially regulate CD. To the best of our knowledge, this is the first study to provide the genetic basis of CD, and the genetic loci identified in this study may ultimately assist in wheat breeding programs.

## 1 Introduction

Bread wheat (*Triticum aestivum* L.) is one of the most important cereal crops. With increasing world population and food consumption per capita, food production needs to increase by 60% to be able to meet the food demands by 2050 (Shiferaw et al., 2013). Therefore, high yield is the long-term, primary, and most important goal of wheat breeding programs, and increasing grain yield is an urgent task to fulfill global food and nutritional security (Li H. et al., 2019).

Wheat yield is one of the important and complex traits, which is controlled by multiple genes and strongly influenced by environmental factors. Thousand grain weight (TGW), spike number per area (SN), and grain number per spike (GNS) are the three main important components of grain yield (Yang et al., 2020). To date, many quantitative trait loci (QTL) associated with grain-related traits have been identified using linkage mapping methods and genome-wide association study (GWAS) (Cao et al., 2020; Yang et al., 2021). However, due to the limitations such as low resolution, low marker density, and unstable results, only a few causative genes were identified and stable QTL were used in practical wheat breeding programs. In the past few years, the reference genome and pangenome of bread wheat were successively released, which greatly promoted research in wheat genomics and population genetics (IWGSC et al., 2018; Walkowiak et al., 2020; Sato et al., 2021; Zhu et al., 2021). Moreover, with the rapid development of genotyping technology, high-throughput genotyping method such as genotyping by sequencing (GBS), exome sequencing, and whole genome resequencing, have been widely used in the genome and population genetic study of wheat (Cheng et al., 2019; He et al., 2019; Zhou et al., 2020). A panel of 768 wheat cultivars that were genotyped using GBS was used to conduct a GWAS; 273 QTL were detected that were associated with 12 traits within 1.0 Mb intervals. Among them, eight putative candidate genes underlying three QTLs were identified, which were also validated in biparental populations (Pang et al., 2020). Recently, exome sequencing was performed on a set of 287 accessions to study the molecular diversity of the wheat mini-core collection, and 6.7% of the wheat genome that underwent asymmetric selection during wheat breeding was analyzed. Moreover, GWAS and gene function analysis revealed that two pleiotropic genes, *TaARF12* and *TaDEP1*, which regulate both plant height and grain weight, play a role in epistasis with the Green Revolution gene *Rht-1* (Li et al., 2022). In addition, a total of 145 Chinese elite wheat cultivars with historical diversity were resequenced, and a whole-genome genetic variation map was depicted (Hao et al., 2020). The study systematically analyzed the formation and evolution of excellent germplasm resources and major varieties during 70 years of wheat breeding in China and indicated that haplotype block dynamics guide genomic-driven selective breeding.

Wheat grain has a complex structure. The pericarp, seed coat, nucellar epidermis, aleurone layer, and starchy endosperm are present from the outside to inside. The embryo is embedded in the seed coat on the dorsal side of the wheat grain (Stomph et al., 2011; De Brier et al., 2016). Compared with the grains of other grass crops, such as rice and maize, wheat grain has a crease on the ventral side, which extends almost to the center of the grain (Sun et al., 2007). The crease connects the tissues of the placenta and chalaza and results in a fused zone that extends along the ventral side of the grain (Frazier and Appalanaidu, 1965; Aziz, 1972; Bechtel et al., 2009). The differentiation of transfer cells occurs in the crease region and nucellar projection throughout the early stages of caryopsis development (Drea et al., 2005). The transfer tissues from the crease region mainly include maternal and endosperm transfer tissues which both play an important role during endosperm and embryo development. The maternal transfer tissues include vascular bundle, chalaza, and nucellar projection transfer cells while endosperm transfer tissues include aleurone transfer cells, starchy endosperm transfer cells, and endosperm conducting cells (Yu et al., 2015). During the development of wheat grain, nutrients are transported mainly via the vascular bundle (Zee and O’brien, 1970; Zheng et al., 2014). Thus, the crease region of wheat is reported as a key organ for regulating flow of mineral micronutrients in the grain (Kamaral et al., 2022), and the function of loading of mineral micronutrients into the endosperm is restricted to specialized cells in the crease region (Ajiboye et al., 2015). Moreover, the depth of crease is also an important determinant of milling yield where kernels with a shallow crease have higher milling yield (Mabille and Abecassis, 2003). However, to date, no study has reported the relationships between the crease depth and grain yield in wheat.

In this study, resequencing and population genomic analysis was performed using 413 accessions, which included 86 landraces and 325 cultivars. The relationship between CD and agronomic traits was identified. Some potential selective footprints were identified by comparing genome-wide genetic diversity between landraces and cultivars. Furthermore, GWAS was conducted to identify the genetic loci and candidate genes associated with CD. To the best of our knowledge, this is the first study to provide the genetic basis of use of CD in breeding improvement, and the genetic loci identified in this study may ultimately assist in improving grain yield in wheat.

## 2 Materials and methods

### 2.1 Plant materials and field trials

In this study, 413 wheat accessions, which included 86 landraces and 325 cultivars, from various regions in the world were selected as the experimental materials. This comprised of 275 accessions from the major agro-ecological zones in China and 137 accessions from other countries. The origin information of remaining accession was not available (**Table S1**).

All populations were planted in the field nursery at Xiangyang (Hubei Province, 32.17°N, 112.13°E) in 2019–2020 (XY19) and Luoyang (Henan Province, 34.82°N, 112.44°E) in 2020–2021 (LY20). Each accession was space-planted in a 3-m single-row plot with 5-cm distance between plants and 20-cm distance between rows. The field experiments used a randomized complete block design with two replicates and standard agronomic wheat management practices were conducted.

### 2.2 Phenotypic evaluation

To evaluate the crease depth trait, the length of real crease depth (L1) and thickness of the grain (L2) were measured (Le et al., 2019; Huang et al., 2022). The seeds were not uniform in size; thus, the ratio of L1 and L2 was used to describe the degree of crease depth in this study (**Figure S1**). The protocol for obtaining the phenotypic data for CD trait was as follows: (1) 30 plump and uniform seeds were randomly collected from each variety (less than 30 seeds were collected for some varieties); (2) all kernels were cut from the middle of the kernel with the assembled security blade, further, the obtained kernel slices were trimmed well; (3) the grain slices were arranged neatly, and pictures were taken using a Canon digital camera; (4) image datasets were imported into a graphical user interface program, which was written using a custom MATLAB script, and the CD value was manually measured; (5) the measured pictures and data were saved. The mean crease depth of 30 grain for each accession was used for subsequent analysis.

Phenotyping of grain-related traits, including TGW, grain length (GL), and grain width (GW), were analyzed using an automatic seed size analyzing system (SC-G, Wanshen Technology Company, Hangzhou, China) from the seeds dried after harvesting.

The quality-related traits, such as grain protein content (GPC), wet gluten content (WGC), water absorption (WA), and stability time (ST), were measured using near infrared transmittance spectroscopy (NITS, Unity Scientific, USA). For each variety, 100 g seeds were weighed and ground using a flour mill. The extraction rate of flour (ER) was calculated as follows: ER = (flour weight/grain weight) × 100% (Naraghi et al., 2019).

### 2.3 Statistical analysis

Basic descriptive statistical analysis and two-tailed t-test were performed using IBM SPSS statistics version 20.0. Pearson’s correlation coefficients between different environments or different traits were calculated using the R base function cor (), and the results with the histogram plot and correlation coefficients were displayed using the PerformanceAnalytics package (https://github.com/braverock/PerformanceAnalytics). The value of the best linear unbiased prediction (BLUP) for trait across multiple environments and broad-sense heritability (*H^2^*) were calculated using lme4 package in R 3.6.1 (http://www.r-project.org/).

### 2.4 Genotyping

Total genomic DNA of all samples was extracted from leaf tissues using the CTAB method, and paired-end sequencing library of each accession was constructed as per the manufacturer’s instructions (Illumina, San Diego, CA, USA). All accessions were sequenced using the MGISEQ-2000 platform. The paired-end reads were mapped to the bread wheat reference genome (IWGSC et al., 2018), and SNP calling for each accession was performed using Sentieon software (Freed et al., 2017). The SNPs with missing rate ≤ 20% and minor allele frequency (MAF) ≥ 5% were filtered. In total, 9,665,188 polymorphic SNPs was retained and used for subsequent analysis. The genome sequencing data for 413 wheat accessions, including the data from 288 accessions generated previously (Gao et al., 2023) and 125 accessions in this study, have been deposited in the public database of the China National Genebank (https://db.cngb.org/cnsa) under the accession number of CNP0004251.

### 2.5 Population genetic analysis

To detect the regions under selection between landraces and cultivars, the diversity ratio (π_landraces_/π_cultivars_) and fixation index (Fst) of different populations with the same sliding-window approach (200 kb windows and 100 kb steps) across the genome were calculated using VCFtools software (Danecek et al., 2011). Furthermore, the cross-population comparison statistical method, namely, XP-CLR (Chen et al., 2010), was employed to scan the whole-genome-level selection signatures between the two subpopulations. Finally, the windows in the top 5% of π ratio, Fst, and XP-CLR scores were considered as the candidate selective regions.

### 2.6 Genome-wide association study

Association analysis was performed using the Fast-LMM program with a mixed linear model (Lippert et al., 2011). BLUP values and single-environment values were applied to GWAS analysis. Considering the potential risk of type II error and combining the GWAS results in this study, a less stringent criterion (*P*-value < 1 × 10^−5^) was selected to detect the significant loci of interest. According to previous studies, the regions 3-Mb upstream and downstream of the significant SNPs could be defined as QTLs and overlapping QTLs were treated as the same QTL (Pang et al., 2020; Li et al., 2022). SNPs with the minimum *P*-value in the QTL were regarded as peak SNPs. Moreover, to improve the reliability of the results of GWAS, GWAS signals (SNPs/QTLs) detected in at least two environments and the GWAS *P*-value of peak SNPs < 6.20 × 10^−7^ (1/the number of independent SNPs; the modified Bonferroni correction was used to control the genome-wide type I error rate) were retained and subjected to subsequent analysis.

### 2.7 Expression profile and gene ontology analysis

The expression data of all candidate genes in the root, stem, leaf, spike, and grain, were downloaded from WheatOmics 1.0 (Ma et al., 2021). Cluster analysis of these candidate genes was carried out using R package Mfuzz (Kumar and Futschik, 2007). Moreover, the gene ontology (GO) enrichment analysis was performed using AgriGO webserver (version 2.0). GO terms with the FDR threshold of 0.05 were selected as significant terms using Fisher’s exact test. To visualize the expression data, heatmap was constructed using TBtools (Chen C. et al., 2020).

### 2.8 Candidate gene identification

To narrow down the range of candidate genes associated with CD, the genes located within QTL regions were extracted from the gene annotations of the reference genome of Chinese Spring (IWGSC Refseq1.1). Meanwhile, homologous genes in *Arabidopsis* and rice were acquired using the protein sequence of these putative genes via the BLASTP program. Genes with annotations related to spike or grain development were regarded as the promising genes. Furthermore, gene expression profiles in the transcriptome data of these genes were examined, and the genes that were predominantly expressed during the spike or grain development stage of wheat were selected as the candidate genes.

## 3 Results

### 3.1 Phenotypic variation of CD and other agronomic traits

**Figure 1:**
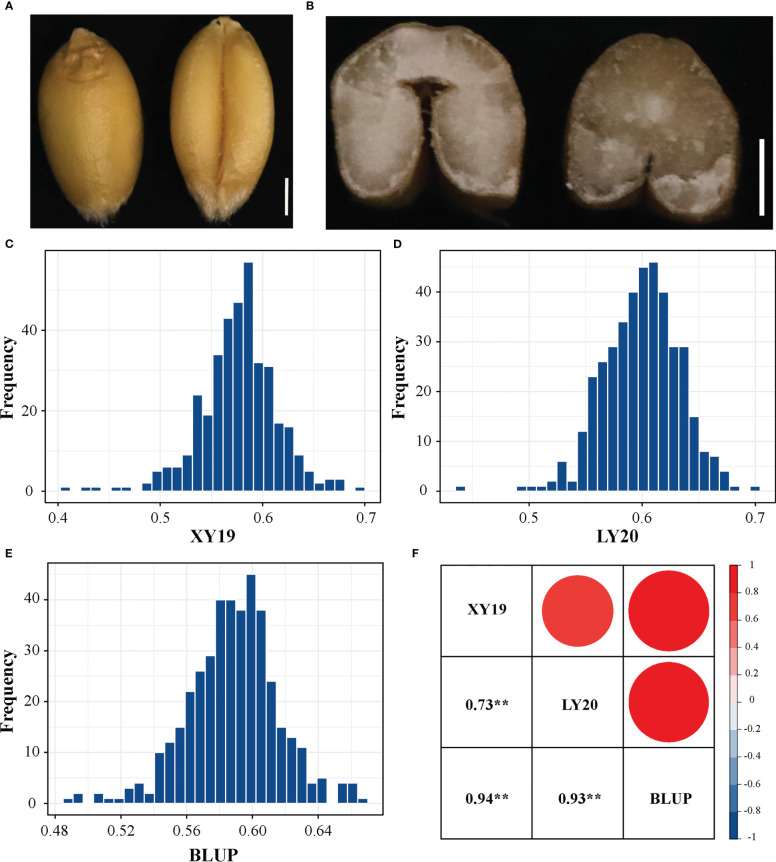
Crease depth (CD) variation in 413 wheat accessions. **(A)** The dorsal and ventral side of wheat grain. Scale bar, 1mm. **(B)** Phenotypes of crease depth in different varieties. Scale bar, 1mm. **(C)** Distribution of CD values in XY19. **(D)** Distribution of CD values in LY20. **(E)** Distribution of CD values in BLUP. **(F)** Correlations among various field environments. XY, Xiangyang; LY, Luoyang; 19 and 20 represent years 2019 and 2020, respectively. BLUP represents the best linear unbiased prediction values across various environments. **P < 0.01 (two-tailed).

Statistical analysis revealed that the phenotypic value of CD in the 413 accessions in all tested environments exhibited continuous and significantly broad variation from 0.404 to 0.699 (**Figures 1**, **S2** and **Table 1**). The mean values of CD were 0.577, 0.600, and 0.588 for XY19, LY20, and BLUP, respectively. The coefficient of variation (CV) of CD ranged from 4.69% (BLUP) to 6.58% (XY19), showing a high degree of dispersion. Moreover, correlation analysis of different environments revealed different degrees of positive correlation (ranging from 0.73 to 0.94) among the tested environments (**Figure 1F**). The *H^2^* of CD was 84.09% (**Table 1**), suggesting that this trait was stably inherited and suitable for genetic analysis.

**Table 1:** Phenotype statistics of crease depth trait in the tested population.

Env.	Min	Max	Mean	SD	CV(%)	*H^2^* (%)
XY19	0.404	0.692	0.577	0.038	6.58	84.09
LY20	0.439	0.699	0.600	0.033	5.56	
BLUP	0.486	0.665	0.588	0.028	4.69	

### 3.2 Relationship between CD and other agronomic traits

To explore the relationship between CD and other major agronomic traits, it was investigated that the phenotypes of these agronomic traits including TGW, GL, GW, ER, GPC, WGC, WA, and ST. All traits exhibited abundant phenotypic variation, and significant relationship was observed among different environments (**Tables S2** and **S3**). Additionally, the broad-sense heritability of these investigated traits ranged from 57.63% (ER) to 93.88% (GL) (**Table S2**), suggesting that all tested traits possessed stable phenotypes.

**Figure 2:**
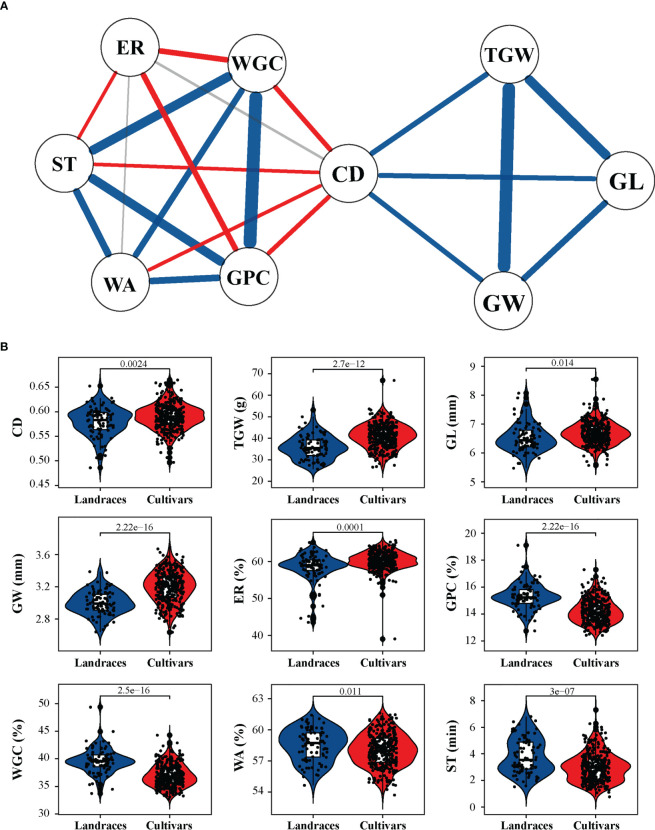
Relationship between crease depth (CD) and other agronomic traits and comparisons between landraces and cultivars with respect to tested agronomic traits. **(A)** Correlation-based network analysis between CD and other traits. The width of the lines represents the strength of correlation between two traits. Blue, red, and gray lines represent positive, negative, and no correlations, respectively. **(B)** Differences between landraces and cultivars in terms of CD, thousand grain weight (TGW), grain length (GL), grain weight (GW), extraction rate of flour (ER), grain protein content (GPC), wet gluten content (WGC), water absorption (WA), and stability time (ST). *P* values are given based on two-tailed *t*-tests.

Based on the BLUP values calculated across all tested environments, analysis of Pearson’s correlation coefficients among all tested traits showed that CD exhibited a significant positive correlation with TGW (r = 0.27), GL (r = 0.26), and GW (r = 0.25) and a significant negative correlation with GPC (r = −0.21), WGC (r = −0.23), WA (r = −0.16), and ST (r = −0.16) (**Figures 2A** and **S3**). Moreover, among the three grain-related traits, TGW was positively correlated with GW (r = 0.92) and GL (r = 0.62), and GW was also positively correlated with GL (r = 0.34). Among the other five quality-related traits, GPC was positively correlated with WGC (r = 0.96), WA (r = 0.39), and ST (r = 0.60) and negatively correlated with ER (r = −0.30). In addition, TGW and GW showed a significant negative correlation (r ranged from −0.43 to −0.34) with both GPC and WGC (**Figure S3**).

When comparing the differences in different groups of these tested traits, it was observed that cultivars had a substantially higher TGW, GW, and GL than landraces (**Figure 2B**). Consistently, differences in CD were observed between landraces and cultivars, and the latter displayed a higher CD value (**Figure 2B**). Thus, it is speculated that CD may have been targeted by artificial selection simultaneously with yield traits during wheat grain improvement.

### 3.3 Identification of genome-wide genetic diversity and selective sweep signals

**Figure 3:**
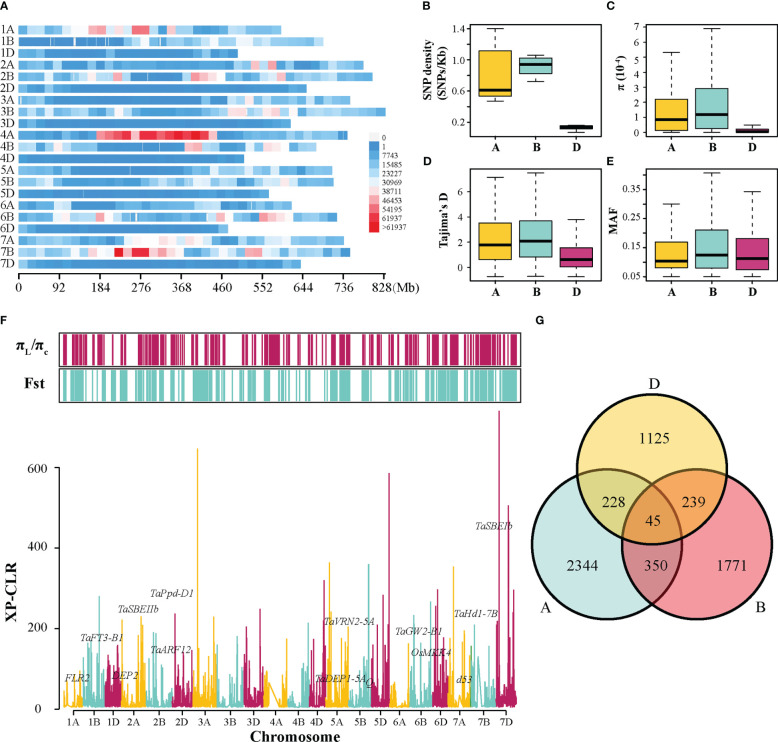
Genetic diversity and selective sweep analysis in the tested population. **(A)** The density distribution of SNPs along 21 chromosomes within 20-Mb window size. The distribution of SNP density **(B)**, π value **(C)**, Tajima’s D value **(D)**, and MAF **(E)** among A, B, and D sub-genomes. **(F)** The distribution of the XP-CLR values along chromosomes, and the selected regions detected by π ratio (π_L/_π_C_) and Fst methods. The genome-wide threshold was defined by the top 5% of the XP-CLR, π ratio, and Fst values. **(G)** Venn diagram of triplet homologous genes under selection.

From 413 resequenced wheat accessions, 9,665,188 high-quality SNPs were filtered (**Figure 3A**). It was observed that A, B, and D sub-genomes contained different numbers of SNPs, with the A, B, and D sub-genomes harboring 4,036,925, 5,077,692, and 550,571 SNPs, respectively (**Table S4**). The most and fewest SNPs were observed on 7B (1,099,579) and 4D (37,147), respectively (**Table S4**). The SNP density varied from 0.07 per kb on chromosome 4D to 1.46 per kb on chromosome 7B, and the overall density in the B sub-genome was 7 times higher than that in the D sub-genome (**Figure 3B** and **Table S4**). Moreover, nucleotide diversity (π), neutral evolutionary parameters (Tajima’s D), and minor allele frequency (MAF) were measured in each sub-genome. The results revealed that the π and Tajima’s D value of the A and B sub-genomes were significantly higher than those of the D sub-genome, and the B sub-genome contained the highest frequent variations (**Figures 3C–E**).

To identify potential genomic regions targeted by selection during wheat improvement, π ratio (π_landraces_/π_cultivars_), Fst, and XP-CLR scores were calculated between landraces and cultivars across 21 chromosomes using SNP data (**Figure 3F**). Using a significance level of the top 5% for these three methods, 5,481 of genome segments with 13,668 genes were detected using at least one method mentioned above. They comprised approximately 15.3% (2,150.8 Mb) of the wheat genome, including 1,035 (carrying 5,775 genes), 756.9 (carrying 4,713 genes), and 358.9 Mb (carrying 3,180 genes) on the A, B, and D sub-genomes, respectively (**Table S5**). Among these selective sweep regions, some functional genes were presumed to regulate grain shape or weight, starch synthesis, heading date, and plant height (**Figure 3F**). Additionally, the asymmetrical selection of genes was investigated using all the triplet genes in the common wheat genome. It was revealed that 2,344, 1,771, and 1,125 genes exhibited a selection signal in the A, B, and D sub-genomes, respectively (**Figure 3G**). A much smaller number of two homoeologous were simultaneously selected, with 350 A and B homoeologous, 228 A and D homoeologous, and 239 B and D homoeologous. Furthermore, only 45 triplets of homoeologous genes were selected simultaneously (**Figure 3G**). These data demonstrated that asymmetric selection occurred among three sub-genomes of wheat during breeding programs.

### 3.4 Association mapping identified potential loci associated with CD

To identify potential loci associated with CD, GWAS was conducted for the panel of 413 wheat accessions under three environments. In total, 551 SNPs were identified that were significantly associated with CD across three environments (**Figure S4** and **Table S6**). The number of SNPs per chromosome ranged from 1 (6B) to 117 (5A), with an average of approximately 26 (**Table S6**). The range of 3 Mb upstream and downstream of the significant SNP was defined as a QTL (Pang et al., 2020; Li et al., 2022). To maximize the credibility of the result of GWAS, only QTLs identified in at least two environments were used for further study. In the end, 18 QTLs were detected in at least two environments in this study (**Table S7**). Similar with the distribution type of SNPs, these QTLs were distributed on various chromosomes as follows: chromosomes 2B (2), 2D (1), 3A (2), 3B (2), 3D (2), 4A (1), 4D (1), 5A (3), 6A (1), 6D (1), 7A (1), and 7D (1). Among them, 83 significant SNPs were observed in QTL12, and the remaining QTL contained less than 20 significant SNPs (**Table S7**).

Moreover, these peak SNPs were verified to be associated with CD in the tested population via *t*-test. Except for SNP-15329560, SNP-23174611, SNP-37715374, SNP-49795956, and SNP-54465571, significant difference (*P* < 0.05) was detected between the phenotypic values of accessions with two alleles at each SNP loci in all tested environments (**Table S8**). Meanwhile, many QTLs in this study overlapped with the selection sweeps and grain-related QTL detected in present or previous studies (**Table S9**). This indicated that these regions were indeed pleiotropic genomic regions for CD and grain-related traits and were simultaneously selected during wheat improvement progress.

### 3.5 Analysis of GO enrichment and expression profiles of candidate genes

**Figure 4:**
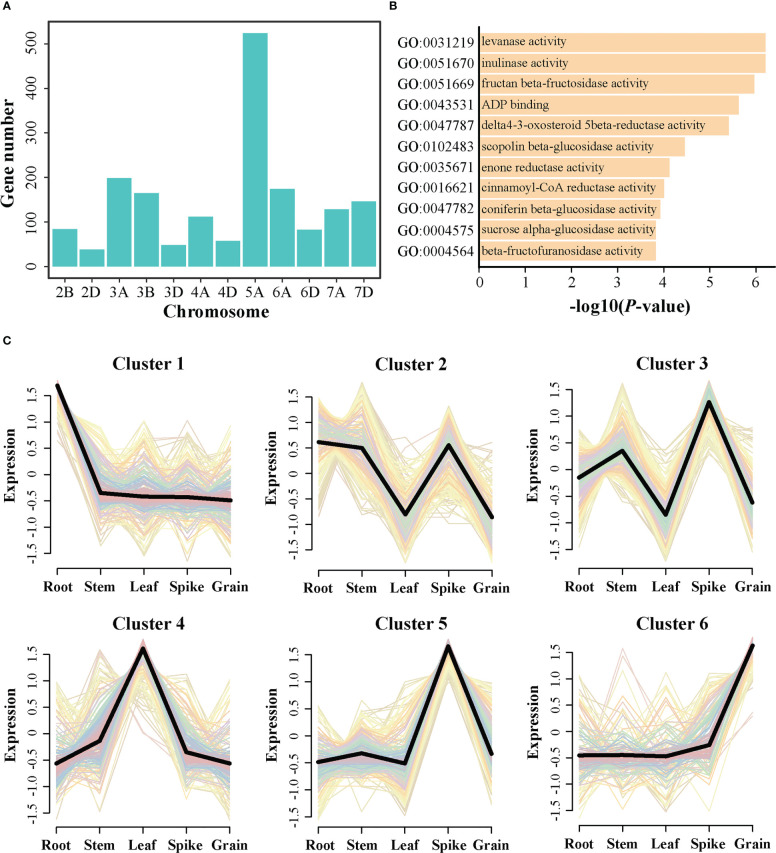
Expression profiles and gene ontology (GO) analysis of the candidate genes identified by genome-wide association study. **(A)** Distribution of the candidate genes on chromosomes. **(B)** The significantly enriched GO terms were identified using GO enrichment analysis. **(C)** The cluster analysis of the candidate genes using the public RNA-seq data.

A total of 1,756 genes were obtained from these identified QTL regions. Further, the distributions of these candidate genes were depicted on the chromosomes (**Figure 4A**). The number of genes detected within the A sub-genome (1,136) was more than that within the B (249) and D (371) sub-genomes. Chromosome 5A had the largest numbers of genes (524), whereas chromosome 2D had only 38 genes. Furthermore, GO enrichment analysis indicated that these genes displayed an enrichment in fructan beta-fructosidase activity (GO:0051669), scopoline beta-glucosidase activity (GO:0102483), coniferin beta-glucosidase activity (GO:0047782), sucrose alpha-glucosidase activity (GO:0004575), and beta-fructofuranosidase activity (GO:0004564) (**Figure 4B**), which may be involved in the growth and development of grain. Additionally, the expression patterns of these genes were explored using the transcriptome data of five main tissues (including root, stem, leaf, spike, and grain). After removing the genes with zero TPM value in all tested tissues, 1,618 genes were finally retained for cluster analysis (**Figure 4C**). The results revealed that these genes could be grouped into six cluster. Cluster 1 included 342 gene highly expressed at root. Cluster 2 contained 235 genes that maintained high expression levels at root, stem, and spike. Cluster 3 included 256 genes with high expression levels at stem and spike. Cluster 4 comprised 247 genes preferentially expressed at leaf. Cluster 5 and Cluster 6 included 348 and 190 genes preferentially expressed in the spike and grain, respectively. Accordingly, the genes from Clusters 3, 5 and 6 would be focused for further analysis, as these genes may be involved in spike or grain development and could be the candidate genes regulating CD.

### 3.6 Identification of candidate genes

**Figure 5:**
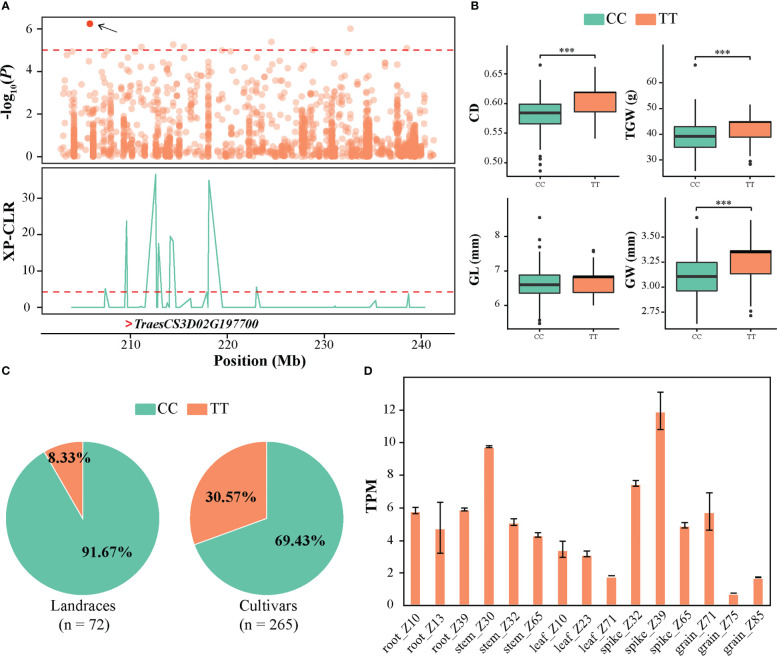
Identification of the crease depth (CD) associated candidate gene *TraesCS3D02G197700* on QTL 3D. **(A)** Local Manhattan plot of 202.81–241.52 Mb genomic region (the genomic interval of QTL9) on QTL 3D (upper). The red point represents the peak SNP (SNP-29314648), and the dashed line indicates the significance threshold (*P*-value < 10^−5^). Selection signal was identified in this region using XP-CLR method (bottom). The “>“ symbol represents the position of *TraesCS3D02G197700*. **(B)** Box plots of CD, TGW, GL, and GW based on the peak SNP allele. The significance levels of the differences were analyzed using two-tailed t-tests. ***P < 0.001. **(C)** The frequency distribution of peak SNP in landraces and cultivars. **(D)** The expression of *TraesCS3D02G197700* in various tissues of wheat retrieved from WheatOmics v1.0.

A stable QTL locus (QTL9) on chromosome 3D from 202.81 to 241.52 Mb was associated with CD, including 10 significant SNPs (**Figure 5A**). Based on the genotype of the peak SNP SNP-29314648, haplotype analysis revealed that the accessions with the alternate TT sequence exhibited higher CD, TGW, GL, and GW than those with the reference CC sequence (**Figure 5B**). Interestingly, it was observed that the ratio of allele TT exhibited an increasing pattern from landrace to cultivar (**Figure 5C**), suggesting that allele TT was preferentially selected as an elite allele in cultivars during wheat improvement. In addition, in this QTL interval, 41 high-confidence protein-coding genes were annotated according to the genome annotations of IWGSC RefSeq v1.1. The expression pattern of the genes in various tissues and stages demonstrated that *TraesCS3D02G197700* exhibited significantly higher expression during spike development, particularly in spike_Z39 (**Figures 5D** and **S5**). Moreover, *TraesCS3D02G197700* was located in the selective sweep region identified above (**Figure 5A**). Its orthologous gene, *OsKinesin-13A*, has been reported to promote microtubule turnover and affect cellulose microfibril orientation and cell elongation via its microtubule depolymerization activity in rice (Tanabe et al., 2007; Kitagawa et al., 2010; Wu et al., 2014). Therefore, it was inferred that *TraesCS3D02G197700* could be the most promising candidate gene underlying QTL9 for CD that contributes to seed development and is involved in artificial selection during wheat breeding programs.

**Figure 6:**
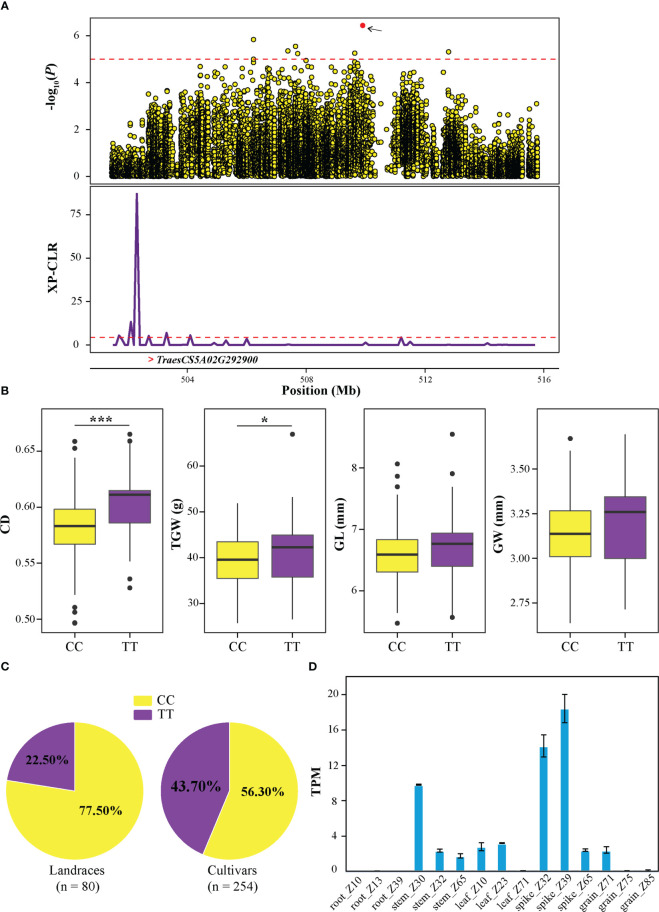
Identification of the crease depth (CD) associated candidate gene *TraesCS5A02G292900* on 5A. **(A)** Local Manhattan plot of genome region 501.45−515.79 Mb (the genomic interval of QTL13) on 5A (upper). The red point represents the peak SNP (SNP-40830175), and the dashed line indicates the significance threshold (*P*-value < 10^−5^). Selection signal was identified in this region using XP-CLR method (bottom). The “>“ symbol represents the position of *TraesCS5A02G292900*. **(B)** Box plots of CD, TGW, GL, and GW based on the peak SNP allele. The significance levels of the differences were analyzed using two-tailed t-tests. ***P < 0.001 and *P < 0.05. **(C)** The frequency distribution of peak SNP in landraces and cultivars. **(D)** The expression of *TraesCS5A02G292900* in various tissues of wheat as retrieved from WheatOmics v1.0.

In addition, another candidate region (QTL13) from 501.45 to 515.79 Mb in chromosome 5A (**Figure 6A**) was further investigated, which contained 9 significantly associated SNPs and 152 candidate genes. Based on the genotype of SNP SNP-40830175, it was observed that TT allele exhibited higher CD, TGW, GL, and GW than CC allele (**Figure 6B**), and the frequency of the allele TT was 22% in landraces and increased to 45% in cultivars (**Figure 6C**). Transcriptome data revealed that *TraesCS5A02G292900* exhibited high expression during spike development stage, particularly in spike_Z39 (**Figures 6D** and **S6**). Interestingly, *TraesCS5A02G292900* falls within the identified selective sweep region. Furthermore, *TraesCS5A02G292900* was a homolog of *OsBC1* that encodes a basic helix-loop-helix (bHLH) transcriptional activator, which has been proven to control cell elongation and positively affect leaf angles in rice (Jang et al., 2017; Jang et al., 2021). These results suggested that *TraesCS5A02G292900* might be involved in grain development and may have undergone selection during wheat breeding process.

## 4 Discussion

CD is the most prominent external morphological feature of wheat grain (**Figures 1A, B**), and the tissues or cells in these regions play an important role in nutrient transportation (Zheng et al., 2014). Because of the gradually decreasing farmland and climate change, increasing grain yield remains the main objective in wheat breeding programs (Li et al., 2018). Among the three factors of grain yield, the heritability of TGW is higher than that of SN and GNS (Li et al., 2018), and TGW has contributed to the wheat yield the most over the past six decades (Wang et al., 2019). Additionally, TGW is determined by kernel dimensions, such as KL and KW (Gegas et al., 2010; Huang et al., 2015). Therefore, a clear understanding of the genetic basis of CD and relationships between CD and grain-related traits may be useful for breeder to improve wheat yield.

### 4.1 CD is largely genetically controlled in wheat

In wheat, grain-related traits are complex quantitative traits controlled by multiple QTLs. Grain-related traits mainly comprise TGW, GW, and GL, and their genetic laws have been studied extensively. Previous studies reported positive correlations among these traits with highly broad sense heritability (Li et al., 2018; Wang et al., 2021). Consistent with previous reports, in this study, TGW, GW, and GL displayed abundant phenotypic variation among tested population, and each trait exhibited a stable phenotype among different environments (**Tables S2**, **S3** and **Figure S3**). Thus, these traits are suitable for genetic research and have been the objective of extensive studies aimed to identify genes controlling target traits. However, as the most notable feature of the grain, the genetic law of CD in wheat remains unknown. In this study, a method was developed to measure the depth of crease and evaluated the CD value of a panel of 413 wheat accessions across multiple environments (**Figure S1**). Large phenotypic variations were observed for CD among the association panel, and the phenotypic distribution of CD was observed to be consistent in all tested environments (**Figures 1C–E**). Moreover, a significant positive correlation was observed among different environments, and CD’s broad-sense heritability was 84.09% (**Table 1** and **Figure 1F**). Thus, these results revealed that CD is a quantitative trait with high heritability and suitable for genetic research.

Further, a correlation-based network analysis was performed to visualize a pattern of association among CD and other agronomic traits (**Figure 2A**). The network analysis revealed that CD was moderately positively correlated with TGW, GL and GW and negatively correlated with quality-related traits, including GPC, WGC, WA, and ST. Furthermore, a negative correlation existed among grain-related traits (TGW and GW) and quality-related traits (GPC and WGC), which was consistent with previous reports (Yao et al., 2018).

### 4.2 Genetic basis of CD

GWAS is reported to be a simple and effective approach for identifying QTLs/genes associated with various agronomic traits; it has been widely used in wheat (Wu et al., 2021; Guan et al., 2022; Shan et al., 2022). As marker density and population size are the two crucial factors affecting the accuracy of GWAS results, sufficiently abundant markers and suitable population sizes are necessary for related studies (Pang et al., 2020). In this study, a panel of 413 wheat accessions was genotyped by resequencing and GWAS was performed to elucidate the genetic basis for CD. A total of 9,665,188 high-quality SNPs were obtained (**Figure 3A** and **Table S4**). It was found that more SNPs were enriched on B subgenome, which was consistent with the previous reports (Hao et al., 2020; Pang et al., 2020; Li et al., 2022). Moreover, the SNP density was approximately 0.69 per kb at whole genome level. This SNP density was higher than that reported by Li et al. (2022) (genotyping by exome sequencing) and less than that reported by Hao et al. (2020) (resequencing for genotyping). This may be mainly due to differences in the choice of genotyping method and sequencing depth.

Moreover, a total of 551 SNPs and 18 stable QTL were observed to be significantly associated with CD via GWAS (**Tables S6** and **S7**). Some QTL overlapped or were close to previously reported grain-related QTL, GWAS signature, or known gene (**Table S9**). For example, *GNI-D1* (Sakuma et al., 2019), *TaVRN2-5A* (Yan et al., 2004), and *KAT-2A* (Chen Y et al., 2020) have been proven to play a significant role in controlling grain weight and were adjacent to the interval of QTL3, QTL14, and QTL15 identified in this study, respectively. In addition, eight QTL, namely, QTL3, QTL4, QTL5, QTL11, QTL12, QTL14, QTL15, and QTL17, were overlapped with the known grain-related QTL, namely, *QTKW-2D-AN* (Mohler et al., 2016), *QTgw-3A1* (Liu et al., 2014), *QTgw.cau.3A_161* (Wang et al., 2021), *QTKW.caas-4DS.2* (Li et al., 2018), *QTKW.ndsu.5A.1* (Kumar et al., 2016), *QTKW.caas-5AL.1* (Li et al., 2019), *QTkw.sdau-6A* (Sun et al., 2009), and *Qmt.tamu.7A.1* (Assanga et al., 2017), respectively. Some previously reported GWAS signatures, such as *KLchr3B143886051* (Hao et al., 2020), *TKWchr3B497701275* (Hao et al., 2020), *TKWchr3D194115132* (Li et al., 2022), and *AX110958315* (Li F et al., 2019), were positioned within QTL6, QTL7, QTL8, and QTL14, respectively. These results together suggested that our results are consistent with previous studies, and that CD-associated genetic segments are pleiotropic and may simultaneously regulate grain-related traits.

### 4.3 CD may undergo artificial selection during wheat breeding

Common wheat is one of the most important crops in the world. It originated in the Fertile Crescent approximately 8000 years ago (Salamini et al., 2002). It has undergone two independent hybridization and polyploidization events and has a hexaploidy genome (He et al., 2019). In order to assess the genetic diversity of the three sub-genomes, the π, Tajima’s D value, and MAF among these sub-genomes were compared. The π and Tajima’s D value of the A and B sub-genomes were significantly higher than those of the D sub-genome, and the B sub-genome contained higher frequent variations (**Figures 3B–E**). This is consistent with previous studies reporting that both A and B sub-genomes underwent a transition from diploid to tetraploid, whereas the D genome did not (Hao et al., 2020).

Long-term natural and artificial selections played a crucial role in wheat domestication and improvement (Pont et al., 2019). Consistent with earlier reports (Li et al., 2022), it was observed phenotypic selection in the tested panel for these agronomic traits during the wheat breeding process, which resulted in the improvement of TGW, GW, and GL. Difference in CD phenotype was investigated among landraces and cultivars, and interestingly, a moderately positive correlation existed between CD and grain-related traits (TGW, GW, and GL). Thus, it was assumed that CD may have undergone unintended selection when grain-related traits are selected during wheat improvement programs. Moreover, the genome-scale selective sweep regions were identified, and some functional genes identified in wheat or those homologous to rice genes were found, such as *TaGW2-B1* (Zhang et al., 2018), *TaDEP1-5A* (Li et al., 2022), *TaPpd-D1* (Beales et al., 2007), *d53* (Itoh et al., 2004), and *Q* (Faris et al., 2003) were reported to regulate grain shape or weight, heading date, and plant height and were selected during wheat breeding (**Figure 3F**). Satisfactorily, all QTLs identified above were overlapped with those selection sweeps identified in this or other previous studies (Hao et al., 2020; Li et al., 2022), indicating that these regions were indeed targets in wheat breeding. Thus, CD may be one of the selection goals in wheat breeding.

### 4.4 Candidate genes for CD

To identify the genes controlling CD in wheat germplasm, a combined analysis of GWAS and gene expression profile was conducted, which has been proven to be a useful approach to explore potential causal genes for complex traits (Schaefer et al., 2018; Sekhon et al., 2019). In this study, 1,756 genes were identified in these QTL regions, and most of the genes were located on A sub-genome, especially chromosome 5A (**Figure 4A**). This phenomenon is similar to previous reports that yield-related QTL-rich clusters were enriched in wheat sub-genome A (Cao et al., 2020). Moreover, enrichment analysis revealed that these genes were enriched in sugar-related pathways (**Figure 4B**). As previously reported, genes that regulate grain development are mainly expressed in spikes or grains, such as *GS5* (Ma et al., 2016), *TaBT1* (Wang et al., 2019) and *TaSPL14* (Cao et al., 2023). Cluster analysis of gene expression profile revealed that the genes in clusters 3, 5 and 6 preferentially expressed at spike and grain developments (**Figure 4C**). Additionally, integrating selective sweep, haplotype and gene expression analysis, two candidate genes (*TraesCS3D02G197700* and *TraesCS5A02G292900*) were identified (**Figures 5** and **6**). *TraesCS3D02G197700* encodes a kinesin-like protein. In rice, its orthologous gene *OsKinesin-13A* plays an important role in promoting microtubule turnover, affecting cellulose microfibril orientation and cell elongation (Tanabe et al., 2007; Kitagawa et al., 2010; Wu et al., 2014). Similarly, the orthologous gene of *TraesCS5A02G292900* in rice, namely, *OsBC1*, affects grain size (Jang et al., 2017; Jang et al., 2021). From the above results, it was inferred that *TraesCS3D02G197700* and *TraesCS5A02G292900* may be the candidate genes of QTL9 and QTL13, respectively, contributing to CD phenotype in wheat.

## 5 Conclusion

In summary, a method was developed for evaluating the crease depth of wheat and the genetic basis of CD was unraveled for the first time. Around 10 million SNPs generated by whole genome resequencing of 413 wheat accessions were used for GWAS. In total, 551 significant SNPs enriched in 18 QTLs were detected significantly associated with CD. Two promising genes *TraesCS3D02G197700* and *TraesCS5A02G292900* were identified as strong candidate genes for CD regulation. To the best of our knowledge, this is the first study to perform the genetic basis of CD, uncovering the potential CD artificial selection together with grain yield during wheat breeding processes. The genetic loci identified in this study may ultimately assist in improving grain yield in wheat.

## Data availability statement

The data presented in the study are deposited in the China National Genebank repository, accession number CNP0004251.

## Author contributions

HM and LG conceived and designed the research. CS, KX, XH, ZZ, AL, YZ and JD performed the experiments. JJ and LG prepared the materials. CS and KX analyzed the data and wrote the manuscript. HM, LG, and JJ revised the manuscript. All authors contributed to the article and approved the submitted version.

## Supplementary material

Supplementary Figure 1 Workflow for crease depth phenotyping.

Supplementary Figure 2 Extreme phenotype of crease depth.

Supplementary Figure 3 Distribution of phenotypic variation of investigated traits and correlation coefficients among the tested traits in 413 accessions. CD: crease depth; TGW: thousand grain weight; GL: grain length; GW: grain weight; GPC: grain protein content; WGC: wet gluten content; WA: water absorption; ST: stability time; ER: extraction rate of flour. * and ** indicate P < 0.05 and 0.01, respectively.

Supplementary Figure 4 Manhattan and quantile-quantile (Q-Q) plots for crease depth in different environments. Red dash line indicates the threshold of genome-wide significant *P*-value (1×10^−5^). XY, Xiangyang; LY, Luoyang; 19 and 20 represent years 2019 and 2020, respectively. BLUP represents the best linear unbiased prediction values across different environments.

Supplementary Figure 5 The expression profiles of candidate genes in the genomic region of QTL9 among various tissues (root, stem, leaf, spike, and grain). *TraesCS3D02G197700* is marked in red, and the colored bar (white to red) represents the relative expression levels.

Supplementary Figure 6 A heatmap for candidate genes located in the genomic interval of QTL13 across various tissues (root, stem, leaf, spike, and grain). *TraesCS5A02G292900* is marked in red, and the scale bar shows the relative expression levels.
