# Supplementary figures and images for: Genome wide association and haplotype analyses for the crease depth trait in bread wheat (Triticum aestivum L.)
Source: Front Plant Sci. 2023 Jul 3;14:1203253. doi: 10.3389/fpls.2023.1203253 (PMC10350514; doi:10.3389/fpls.2023.1203253)

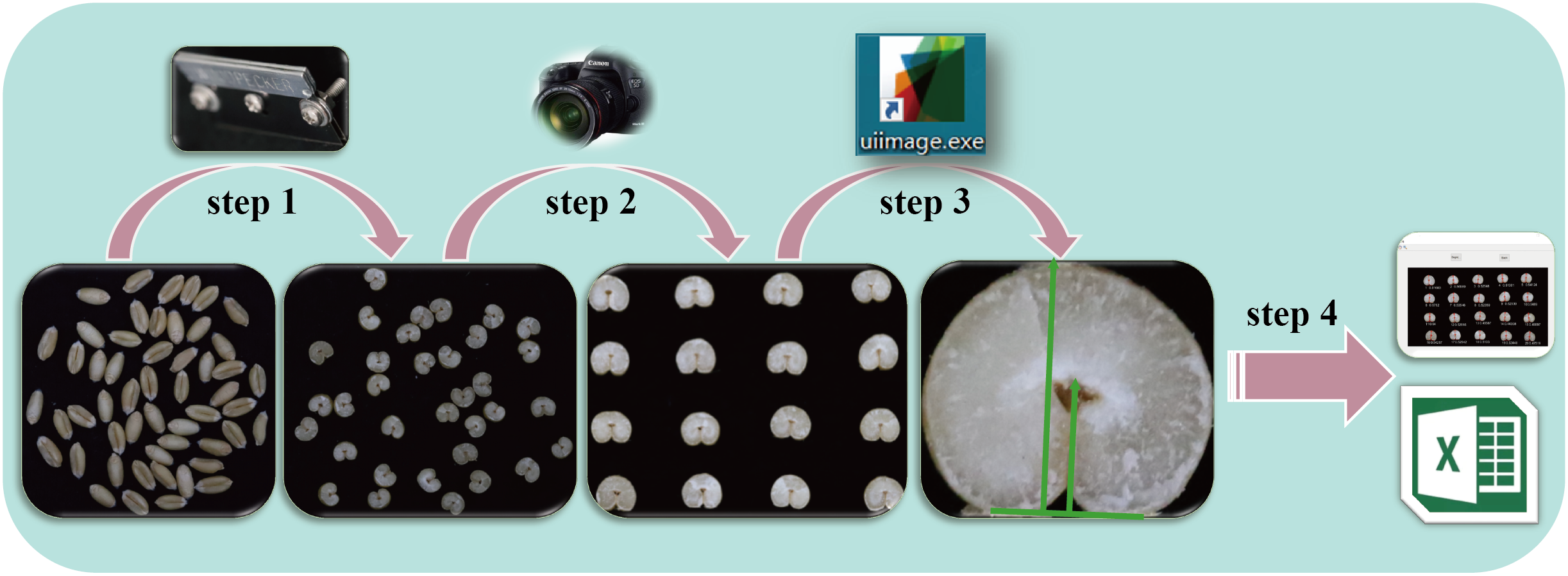

Supplement: Supplementary Figure 1 — Workflow for crease depth phenotyping. [file Image_1.tif]

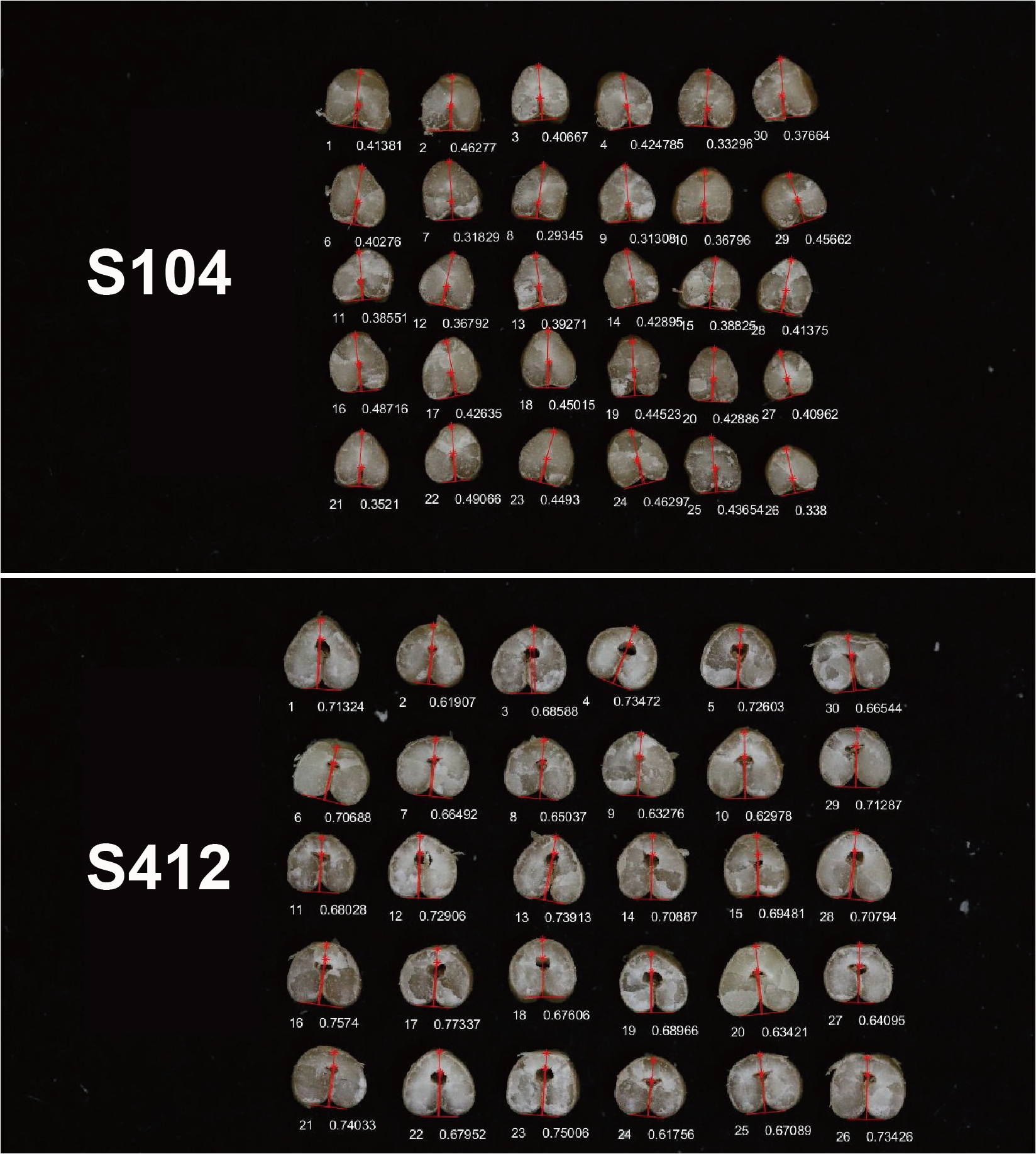

Supplement: Supplementary Figure 2 — Extreme phenotype of crease depth. [file Image_2.tif]

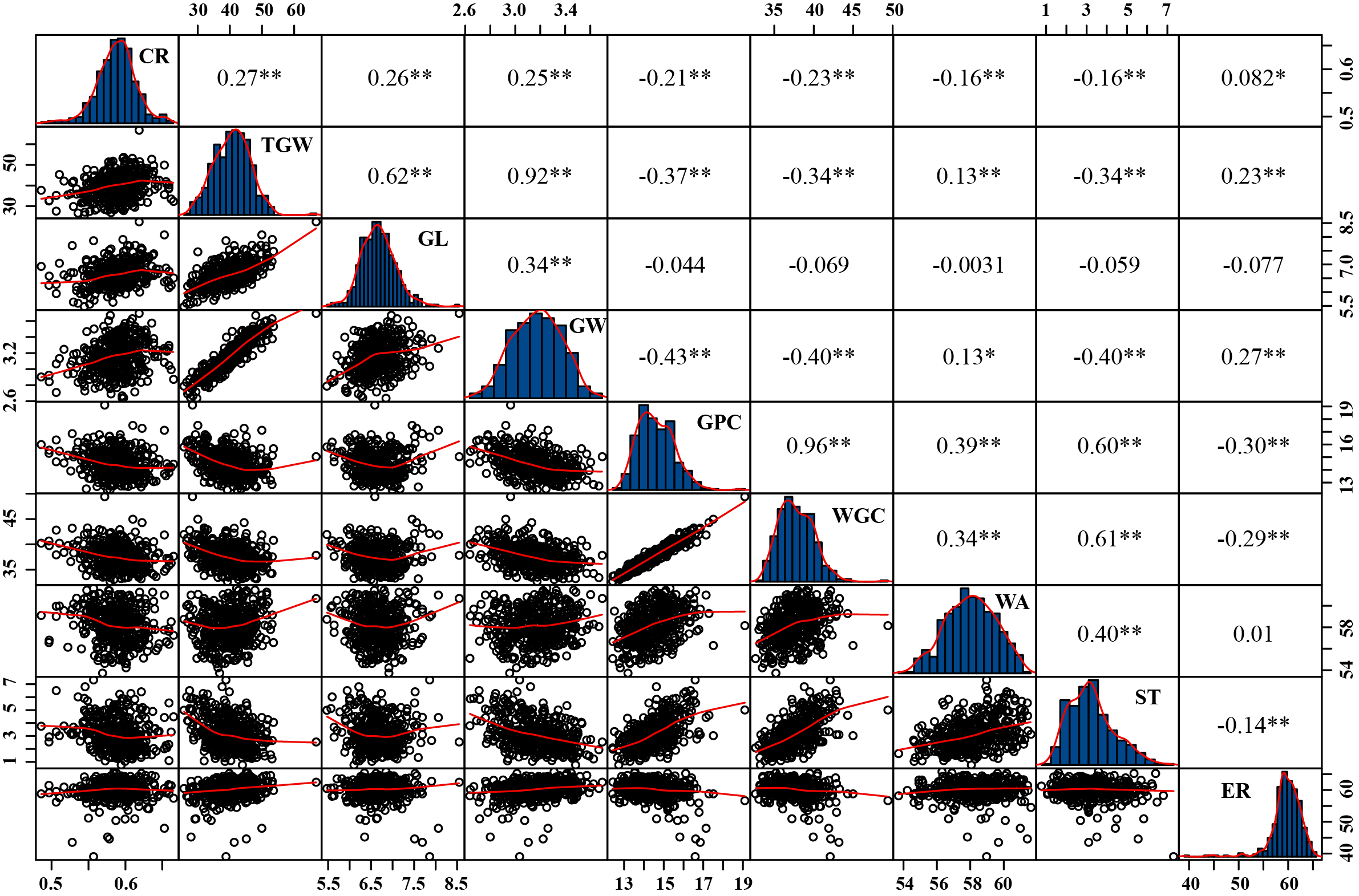

Supplement: Supplementary Figure 3 — Distribution of phenotypic variation of investigated traits and correlation coefficients among the tested traits in 413 accessions. CD: crease depth; TGW: thousand grain weight; GL: grain length; GW: grain weight; GPC: grain protein content; WGC: wet gluten content; WA: water absorption; ST: stability time; ER: extraction rate of flour. * and ** indicate P < 0.05 and 0.01, respectively. [file Image_3.tif]

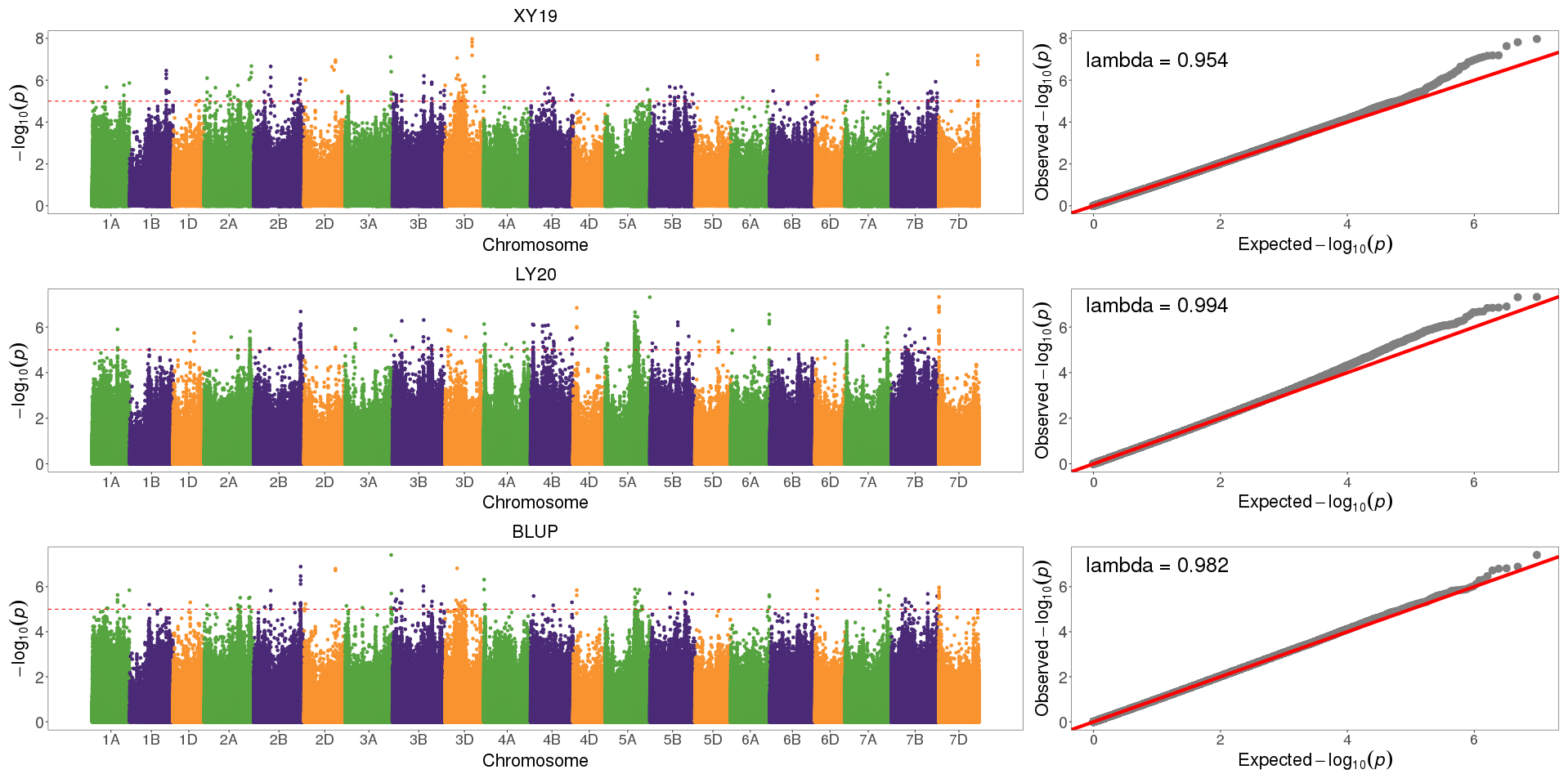

Supplement: Supplementary Figure 4 — Manhattan and quantile-quantile (Q-Q) plots for crease depth in different environments. Red dash line indicates the threshold of genome-wide significant P-value (1×10^−5^). XY, Xiangyang; LY, Luoyang; 19 and 20 represent years 2019 and 2020, respectively. BLUP represents the best linear unbiased prediction values across different environments. [file Image_4.tif]

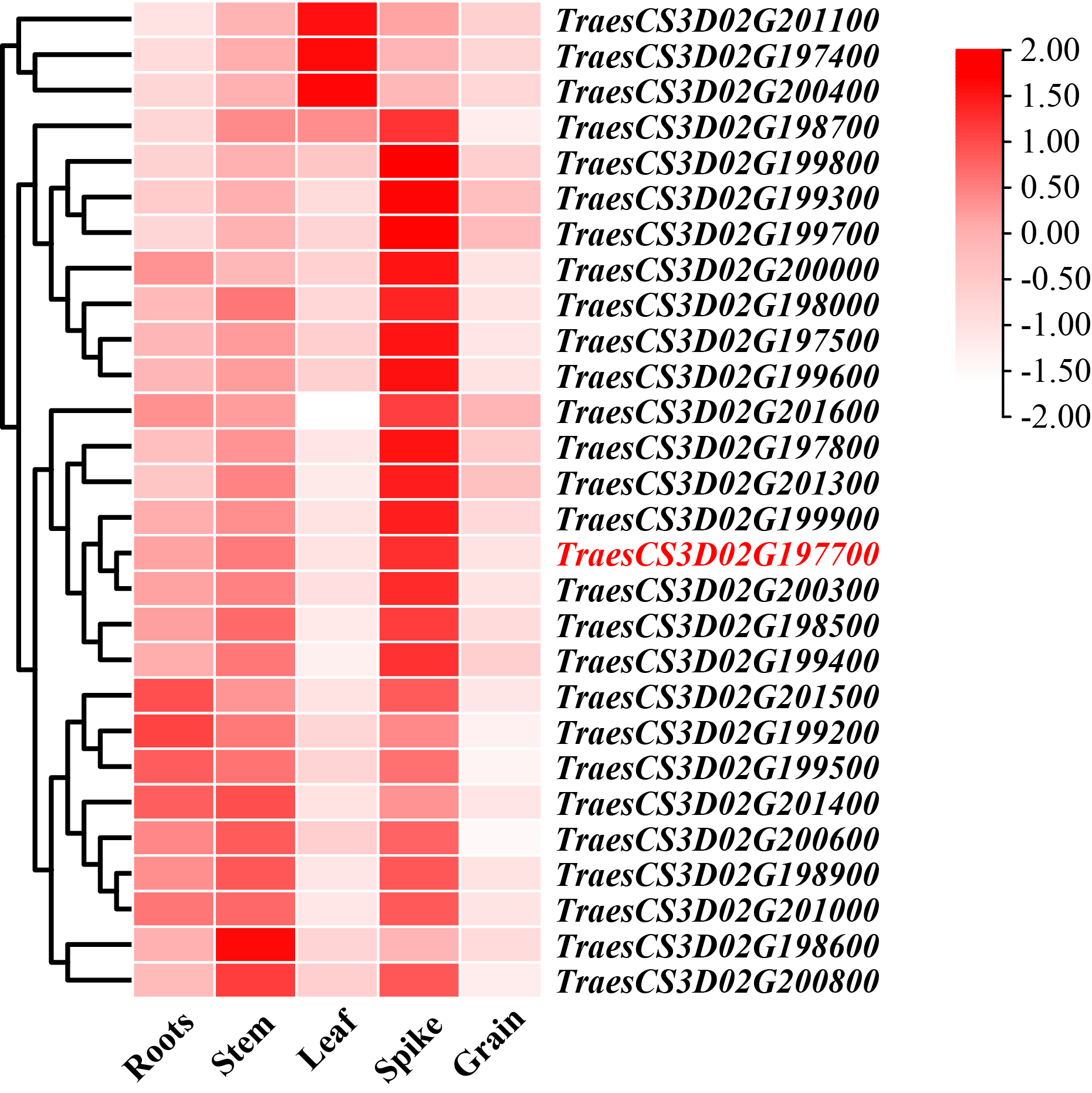

Supplement: Supplementary Figure 5 — The expression profiles of candidate genes in the genomic region of QTL9 among various tissues (root, stem, leaf, spike, and grain). TraesCS3D02G197700 is marked in red, and the colored bar (white to red) represents the relative expression levels. [file Image_5.tif]

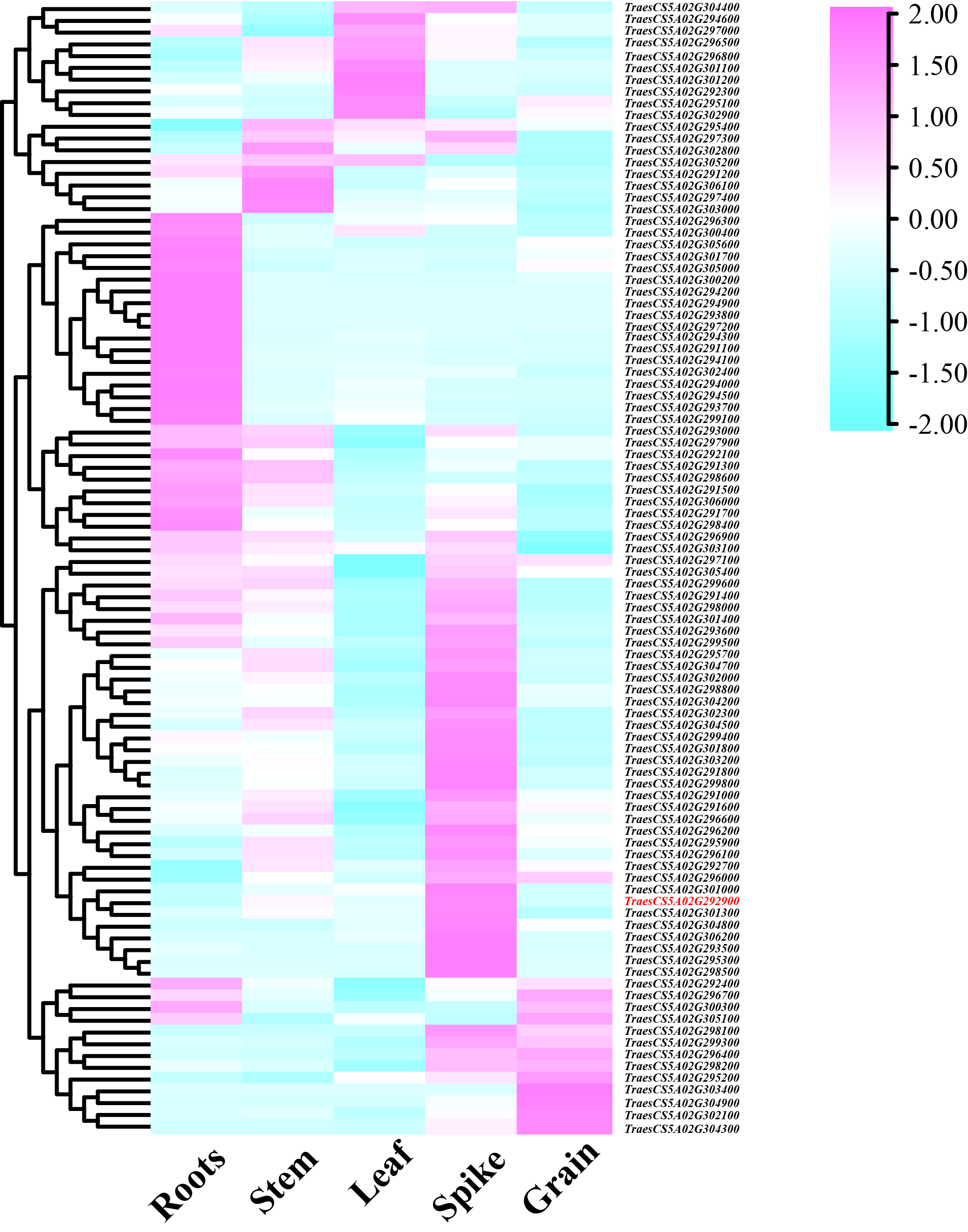

Supplement: Supplementary Figure 6 — A heatmap for candidate genes located in the genomic interval of QTL13 across various tissues (root, stem, leaf, spike, and grain). TraesCS5A02G292900 is marked in red, and the scale bar shows the relative expression levels. [file Image_6.tif]
